# 3D microfluidic *in vitro* model and bioinformatics integration to study the effects of Spatholobi Caulis tannin in cervical cancer

**DOI:** 10.1038/s41598-018-29848-y

**Published:** 2018-08-16

**Authors:** Nijia Wang, Jiayi Wang, Xiansheng Meng, Yongrui Bao, Shuai Wang, Tianjiao Li

**Affiliations:** 1 School of Pharmacy, Liaoning University of Traditional Chinese Medicine, Dalian, 116600 China; 2 Component Medicine Engineering Research Center of Liaoning Province, Dalian, 116600 China; 3 Liaoning Province Modern Chinese Medicine Research Engineering Laboratory, Dalian, 116600 China; 4 Liaoning University of Traditional Chinese Medicine-Agilent Technologies Modern TCM and Multi-omics Research Collaboration Laboratory, Dalian, 116600 P. R. China; 5 Liaoning Institute for Drug Control, Shenyang, 110036 China

## Abstract

Cervical cancer is considered the fourth most common malignant disease in women. Recently, tannin from Spatholobi Caulis (TTS) has been shown to have potent anticancer and antiproliferative characteristics in a few preliminary studies. This experiment used 3D microfluidic, flow cytometry, and gene chip technology to study the efficacy and mechanism of action of TTS, as well as molecular docking technology to study the effect of drugs on related proteins. The cell survival rates of the five groups measured by the 3D microfluidic chip were 94%, 85%, 64%, 55%, and 42%, respectively. With the increase in drug concentration, the cell survival rate gradually decreased. Apoptosis rates detected in the five groups were 2.12%, 15.87%, 33.40%, 41.13%, and 55.10%, respectively. These data suggest that TTS can promote cell apoptosis. The percentages of cells in the G0/G1 phase were 43.39%, 55.07%, 59.57%, 64.56%, and 67.39% in the five groups, respectively. TTS was demonstrated to inhibit the conversion of cells from G0/G1 to S phase and G2/M phase and inhibit gene and protein synthesis to block cell proliferation. TTS can effectively modulate pathogenic proteins. The results confirmed the efficacy of TTS against HeLa cells and that TTS can be used as an adjunct in cervical cancer prevention and treatment.

## Introduction

Cervical cancer is one of the primary diseases that seriously jeopardizes the health and lives of females, according to concerning related statistics; the mortality rate of cancer has presented an increasing tendency over recent years. Overall, it is the fourth major disease that currently affects women’s health^1^. At present, treatment with chemotherapeutics and surgery is efficacious and advisable in cases of cervical cancer because this approach can quickly be curative. However, the toxicity and tolerability of chemotherapeutic drugs and the surgical trauma are the main challenges of this clinical treatment strategy^2^. The development and application of Chinese medicines with high efficacy and low toxicity is universally beneficial. Many researchers have focused on Chinese medicine to find new types of targeted therapy for cervical cancer^3,4^. Spatholobi Caulis is a Manchu drug in Traditional Chinese Medicine (TCM), as recorded in the Chinese Pharmacopoeia (2015), and the use of decoctions containing Spatholobi Caulis is common. Most people consider it to tonify the blood and arrest or regulate menstrual bleeding. Tannin is an active ingredient with a high content in Spatholobi Caulis. Studies in the literature have shown that tannin plays important roles in many pharmacological mechanisms, such as anti-inflammatory and anticancer processes, including in breast cancer and human colorectal cancer^5–7^. Therefore, Spatholobi Caulis is a promising anticancer drug.

Through this experiment, we confirmed most of the ingredients in TTS using ultra high-performance liquid chromatography coupled to time-of-flight mass spectrometry (UHPLC-Q-TOF/MS) and targeted MS/MS data acquisition strategies. Fourteen compounds were tentatively or positively identified, laying the material foundation for the experiment. Microfluidic technology has emerged in recent years, and its most attractive feature is that it can be used to run tests under conditions similar to the human microenvironment; additionally, it is rapid, sensitive and economical to use^8–10^. To date, most relevant experiments have cultivated cancer cells in a two-dimensional (2D) culture system. These monolayer cancer cell culture models have advantages in terms of time and cost compared to *in vivo* models; nevertheless, these models tend to exhibit different signaling and cellular functions than *in vivo* models and therefore differ from their *in vivo* control groups. Many studies have shown that cells cultured in 2D *in vitro* cannot comprehensively reflect the state of cells in the human body^11,12^. In 3D culture, the cytoskeleton of tumor cells is rearranged, and its structure closely resembles that of human tissues. Therefore, the use of three-dimensional (3D) microfluidic *in vitro* models is a powerful method for screening the effects of pesticides and evaluating the mechanisms of drugs. With better control of microfluidic technology, such techniques could additionally be engineered to confine cells in a more physiological context for the bionic environment. 3D microfluidic *in vitro* models are challenging, and we envision that with their development, biomaterial platforms with controlled 3D structures will prove to be valuable systems. Furthermore, one study has shown that the effects of drugs on the viability of tumor cells could be observed in a 3D environment^13^. The formation of cell clusters was observed by cell staining, and the pharmacological activity and efficacy were observed by fluorescence staining. Based on a 3D culture system, the purpose of this study was to investigate the anticancer activity of TTS in terms of induced cell death.

Based on the results obtained in previous experiments, the mechanism of action is further elaborated. The flow cytometry test results suggest that TTS plays a certain role in inducing apoptosis, with a positive correlation between apoptosis intensity and TTS concentration.

**Figure 1:**
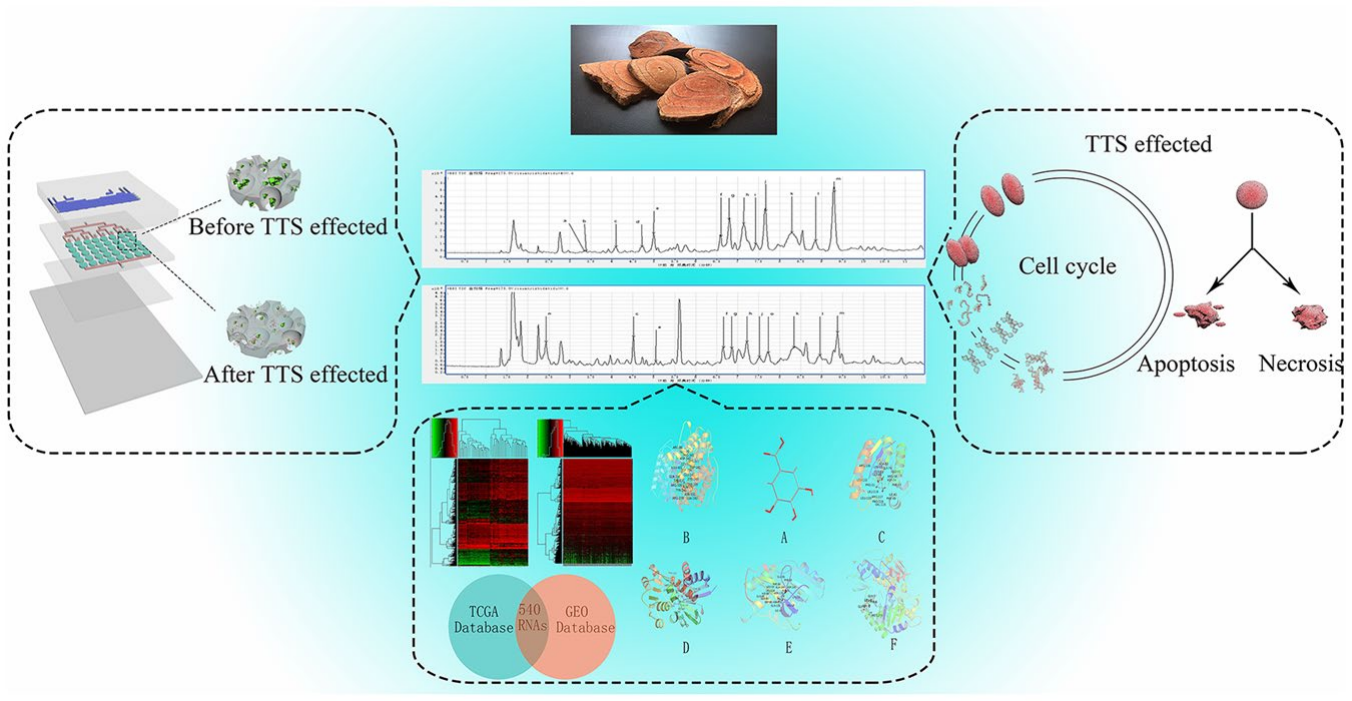
Study design. This study validated the use of TTS in a 3D chip environment against cervical cancer cells using bioinformatics and molecular docking analyses. The effect of TTS on the cell cycle and apoptosis of HeLa cells was also measured. The gray cylinder represents the change in the cell mass in the chip after exposure to the drug.

The experiments showed that the proportions of apoptotic and necrotic cells increased with increasing drug dose compared with the blank group. By arresting cervical cancer cells in the G0/G1 phase, these cells were prevented from transitioning into the S phase, thereby inhibiting proliferation. In this way, DNA and protein synthesis were also inhibited^14^. Gene chips can be used to evaluate the expression of genes related to various biological processes. We looked for differentially expressed RNAs using the Gene Expression Omnibus (GEO) and The Cancer Genome Atlas (TCGA) databases. Genes and proteins were evaluated by Gene Ontology (GO), Kyoto Enrichment of Genes and Genomes (KEGG), and protein-protein interaction network analyses. The effect of tannin on related proteins was simulated by molecular docking. All of the results show that TTS plays an important role in the treatment of cervical cancer. Based on this study of its efficacy and mechanism of action, combined with modern clinical treatment, these results provide a basis for the use of TTS in the treatment of cervical cancer [Fig. 1].

## Results

### Identification of the chemical composition of TTS extracted by UPLC-Q-TOF/MS

**Figure 2:**
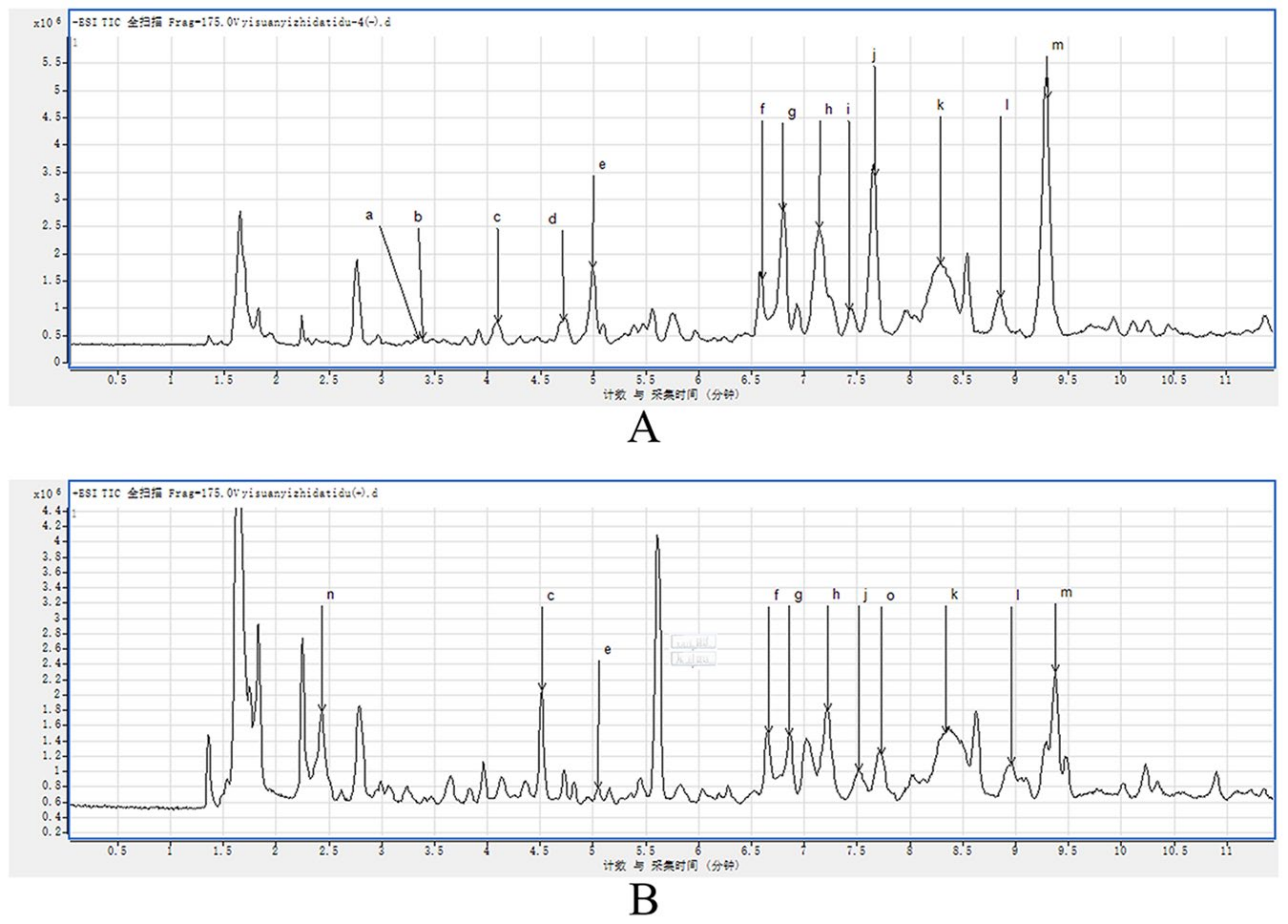
MS chromatograms of TTS. (**A**) Negative ionization mode. (**B**) Positive ionization mode.

In Fig. 2, the base peak ion (BPI) chromatogram of TTS is shown. A total of 14 compounds were tentatively identified based on molecular ions, retention time, major fragment ions, online databases and published articles. The details of the identified compounds are listed in Table 1 ^15–19^.

**Table 1:** Identification of TTS peaks on chromatography profiles in Fig. 3.

Peak No.	CAS number	t_R_	Compounds identification	Molecular formula	m/z		MS/MS
					Calculated	Observed	
a	99-24-1	3.340	Methyl gallate	C_8_H_8_O_5_	184.1461	183.0303	140, 124
b	149-91-7	3.381	Gallic acid	C_7_H_6_O_5_	170.1195	169.0146	146, 137, 125, 119
c	13405-60-2	4.092	β-Glucogallin	C_13_H_16_O_10_	332.26	331.1043	168, 146, 192, 112
d	99-50-3	4.713	3,4-Dihydroxybenzoic acid	C_7_H_6_O_4_	153.1127	153.0194	146, 125, 112, 109
e	3371-27-5	4.994	(−)-Gallocatechin	C_15_H_14_O_7_	306.27	305.0677	261, 219.167, 125, 111
f	20315-25-7	6.599	Procyanidin B1	C_30_H_26_O_13_	577.1287	577.1370	407, 289, 245, 125
g	970-74-1	6.797	(−)-epigallocatechin	C_15_H_14_O_7_	306.2675	305.669	261, 219, 167, 125, 111
h	29106-49-8	7.153	Procyanidin B2	C_30_H_26_O_12_	578.52	577.1370	425, 407, 289, 245, 125
i	139-85-5	7.410	3,4-Dihydroxybenzaldehyde	C_7_H_6_O_3_	138.12	137.0249	112, 108
j	7295-85-4	7.657	Catehin	C_15_H_14_O_6_	289.0521	289.0728	245, 203, 179, 151, 137, 125, 109
k	23567-23-9	8.294	Procyanidin B3	C_30_H_26_O_12_	578.5202	577.1370	531, 451, 407, 289, 273, 161, 137, 125
l	490-46-0	9.295	Epicatechin	C_15_H_14_O_6_	290.27	289.0728	245, 203, 179, 151, 137, 125
m	831-61-8	2.332	Ethyl gallate	C_9_H_10_O_5_	198.17	196.0581	174, 130, 102
n	1257-08-5	7.725	(−)-epicatechin gallate	C_22_H_18_O_10_	442.3723	441.1723	130, 102

### Cell mass growth in chip

**Figure 3:**
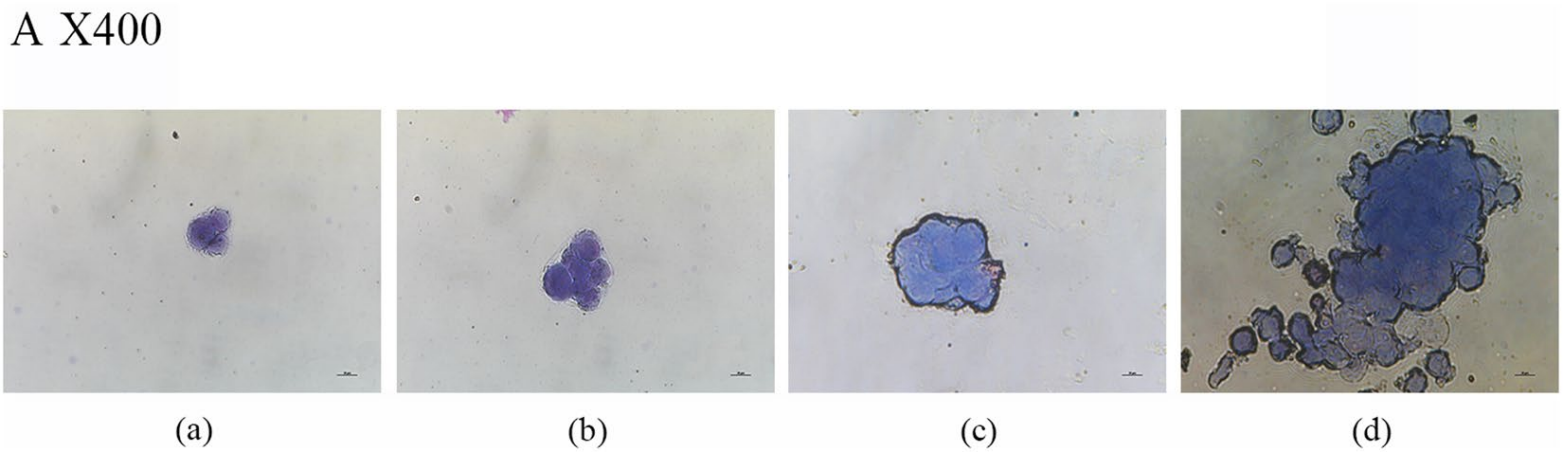
Cell mass growth in chips. Cell mass diameter after growth in the chip for 3, 6, 9, and 12 days; (**a**) 3 days, (**b**) 6 days, (**c**) 9 days, (**d**) 12 days.

Growth of the cell mass within the chip was detected by Wright-Giemsa staining, which stains the cell nucleus blue and the cytoplasm pink. In this experiment, the cells were cultured in the chip for 3, 6, 9, and 12 days. The cell growth status and cell mass diameter in the chip are shown in Fig. 3. The number of cells significantly increased and the cell mass gradually increased over the study period. The cell mass diameter reached up to 135 µm. These results illustrate that PDMS has no toxic effects and that cells can grow successfully in this chip. In summary, this 3D microfluidic culture device can simulate the body environment and provide a beneficial and stable growth environment for cell mass growth.

### Effects of TTS on the cell mass in the chip

**Figure 4:**
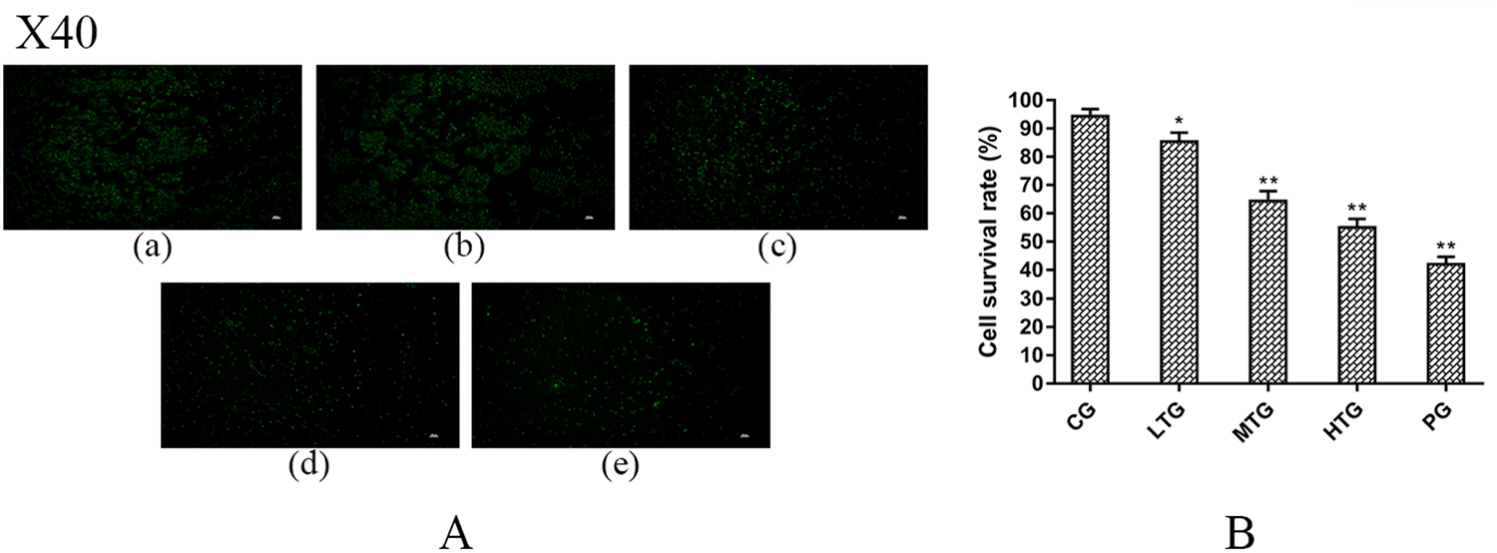
TTS effects on the cell mass in the chip (mean (SD), n = 3). (**A**) Fluorescence images after treatment of cells with different doses of TTS. (a) GC, (b) LTG, (c) MTG, (d) HTG, (e) PG. (**B**) All the cells were counted using IPP (version 6.0) to calculate the cell survival rate (%). The cell survival rate (%) = (normal cells/total number of HeLa cells) ×100% (*P = 0.02, **P < 0.01 vs CG).

The TTS was injected into the chip using a peristaltic pump. After exposure to the drug for 36 hours, the proliferation of HeLa cells was examined using calcein AM and PI staining. As shown in Fig. 4A, the results indicate that exposure to different concentrations of TTS for 36 hours has a significant antitumor effect on HeLa cells; after the drug treatment, the cell mass was significantly reduced, and the intercellular force was weakened. The proliferation of HeLa cells was significantly more inhibited in the treatment groups than in the blank control group, showing a good dose-effect relationship. IPP software was used to analyze the ratio of live to dead cells [Fig. 4B]. With increasing concentration, the proportion of living cells decreased significantly, and with the viability in the high-dose group approaching that in the positive control group (PG). The results show that TTS has latent clinical value against cervical cancer.

### Flow cytometry analysis of apoptosis and the cell cycle

**Figure 5:**
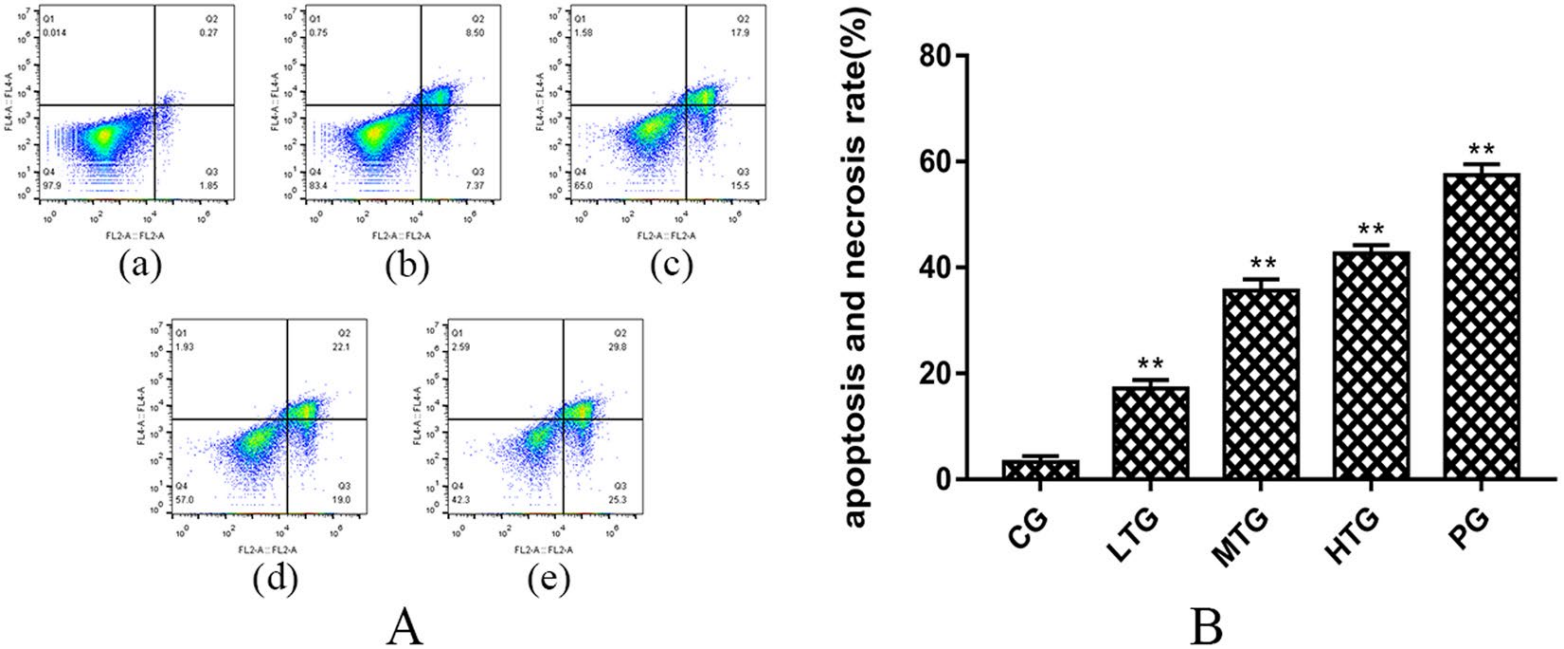
The proapoptotic effect of TTS on HeLa cells (mean (SD), n = 3). (**A**) (a) CG, (b) LTG, (c) MTG, (d) HTG, and (e) PG. (**B**) Histogram of the apoptosis and necrosis rate in each group after treatment with TTS and paclitaxel at different concentrations for 36 hours (**P < 0.01 vs CG).

**Figure 6:**
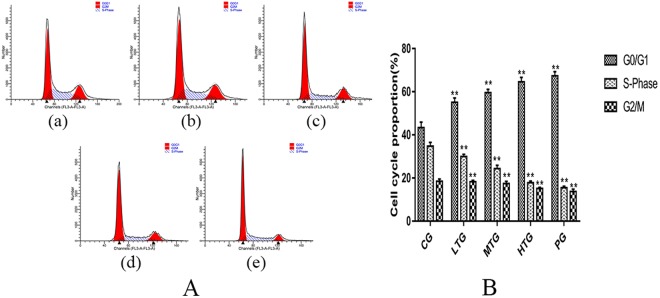
The effect of TTS on the HeLa cell cycle (mean (SD), n = 3). (**A**) (a) CG, (b) LTG, (c) MTG, (d) HTG, and (e) PG. (**B**) Histogram of different cell cycle proportions in each group after treatment with TTS and paclitaxel at different concentrations for 36 hours (**P < 0.01 vs CG).

An Annexin V-FITC/PI Double-Fluorescence Detection Kit was used to determine the effect of TTS on HeLa cell apoptosis, and FlowJo software was used for the analysis [Fig. 5A]. The ModFit software results show that with increasing dose, the percentage of apoptosis gradually increased. The apoptosis and necrosis rate in the low-dose TTS group (LTG) was 15.87 (1.09) % (**P < 0.01 vs CG), while that in the middle- and high-dose TTS groups (MTG, HTG) was 33.40 (1.38) % (**P < 0.01 vs CG) and 41.13 (1.11) % (**P < 0.01 vs CG), respectively. It is noteworthy that the apoptosis and necrosis rate in the PG was similar to that in the HFG at 55.1 (1.37) % (**P < 0.01 vs CG). These data are shown in Fig. 5B. RNase A, PI and Triton X-100 were used to determine the effect of TTS on the cell cycle in HeLa cells, and ModFit software was used for the analysis [Fig. 6A]. The proportion of cells in the G0/G1 phase in the LTG was 55.07 (1.86) % (**P < 0.01 vs CG), while that in the MTG and HTG was 59.57 (1.70) % (**P < 0.01 vs CG) and 64.56 (1.88) % (**P < 0.01 vs CG), respectively. The proportion in the HTG was similar to that in the PG at 67.39 (1.83) % (**P < 0.01 vs CG), and the results in all experimental groups were greater than that in the control group. The proportion of cells in the G0/G1 phase in the HTG was significantly increased compared with that in the CG, while that of cells in the G2/M and S phase was significantly decreased [Fig. 6B]. These results show that TTS inhibits the proliferation of cells by inhibiting both the formation of intracellular DNA and cell mitosis.

### Aberrant gene expression landscape in cervical cancer

**Figure 7:**
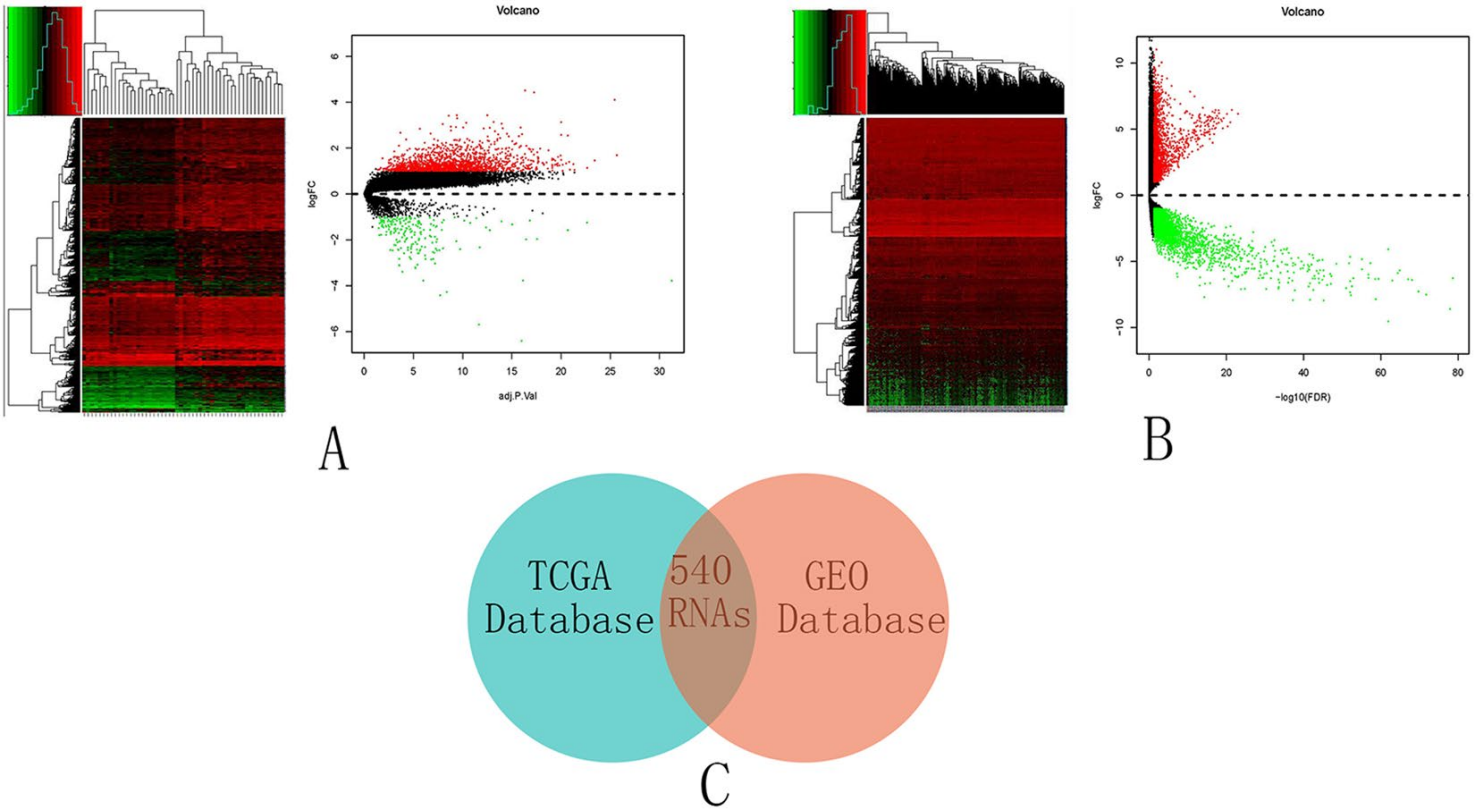
Aberrant gene expression landscape in cervical cancer. (**A**) Differential expression between cervical cancer and normal tissues in the GEO database. (**B**) Differential expression between cervical cancer and normal tissues in the TCGA database. (**C**) The same coding genes were integrated from the GEO and the TCGA databases. Volcano plot of the RNA matrix generated according to TCGA and GEO. Red puncta represent highly expressed RNAs. Green puncta represent downregulated RNAs.

**Figure 8:**
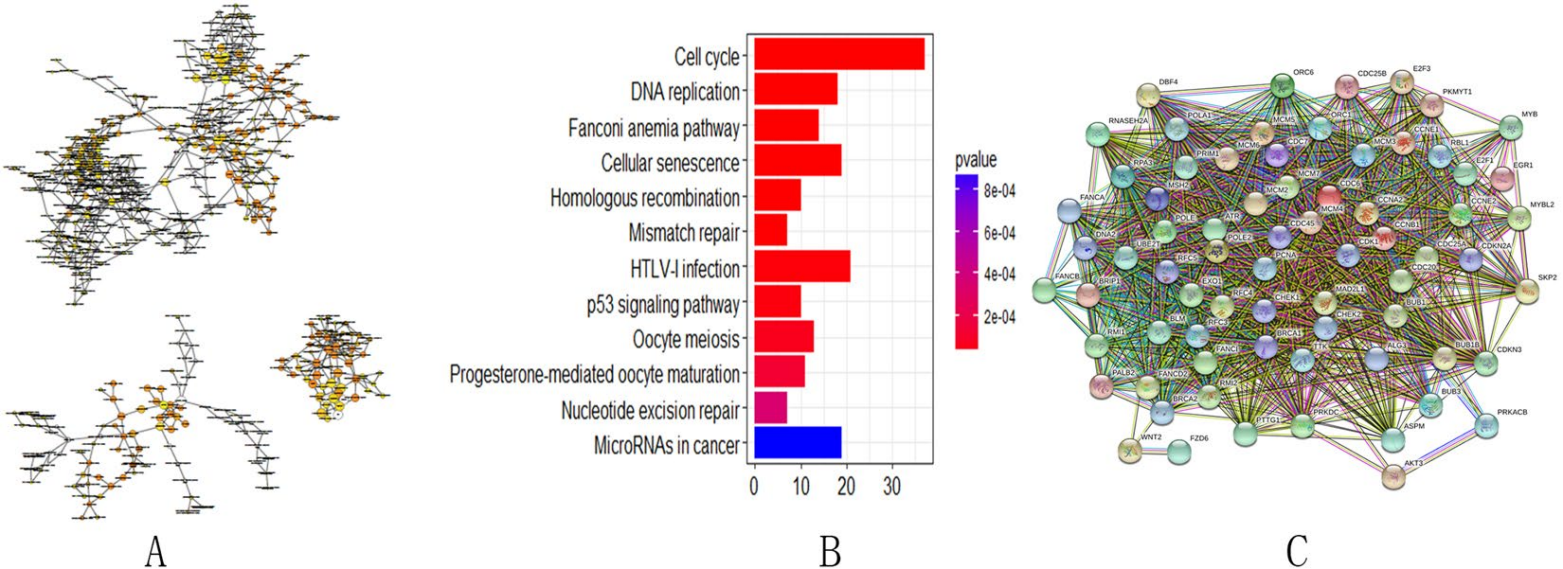
Gene and protein relationships. (**A**) GO enrichment analysis of related genes includes molecular functions, biological processes and cellular components. (**B**) KEGG pathway analysis. (**C**) Protein-protein interaction network of the host genes of these differentially expressed RNAs.

A cohort containing data for 28 cervical cancer patients and 24 normal patients was selected from the GEO database and analyzed. In the cohort (GSE63514), 1904 coding genes showed significant differential expression between the cervical cancer and normal tissues [Fig. 7A]. There were 1699 upregulated RNAs and 205 downregulated RNAs in the cohort. A cohort containing data for 306 cervical cancer patients and 3 healthy subjects was selected from the TCGA database and analyzed. A total of 5025 coding genes showed significant differential expression between the cervical cancer and normal tissues [Fig. 7B]. There were 1971 upregulated RNAs and 3054 downregulated RNAs in the cohort. The same coding genes were integrated from the GEO and the TCGA databases [Fig. 7C]. To explore the possible functions of these RNAs, association analyses were used. KEGG pathway analysis and GO were also investigated for the same identified coding genes. DAVID (https://david.ncifcrf.gov/) and Cytoscape were used to investigate the related biological processes of the enriched genes. For the cell-cell adherens junction, blood vessel endothelial cell proliferation involved in sprouting angiogenesis and cell-matrix adhesion were identified in the GO function analysis [Fig. 8A]. The cell cycle, DNA replication, the Fanconi anemia pathway and the cellular senescence pathway were identified in the KEGG pathway analysis [Fig. 8B]. A protein-protein interaction network of the host genes of these differentially expressed RNAs were also built [Fig. 8C]. The 62 interacting proteins in this network provided a basis for the molecular docking analysis.

### Molecular docking study with tannin

**Figure 9:**
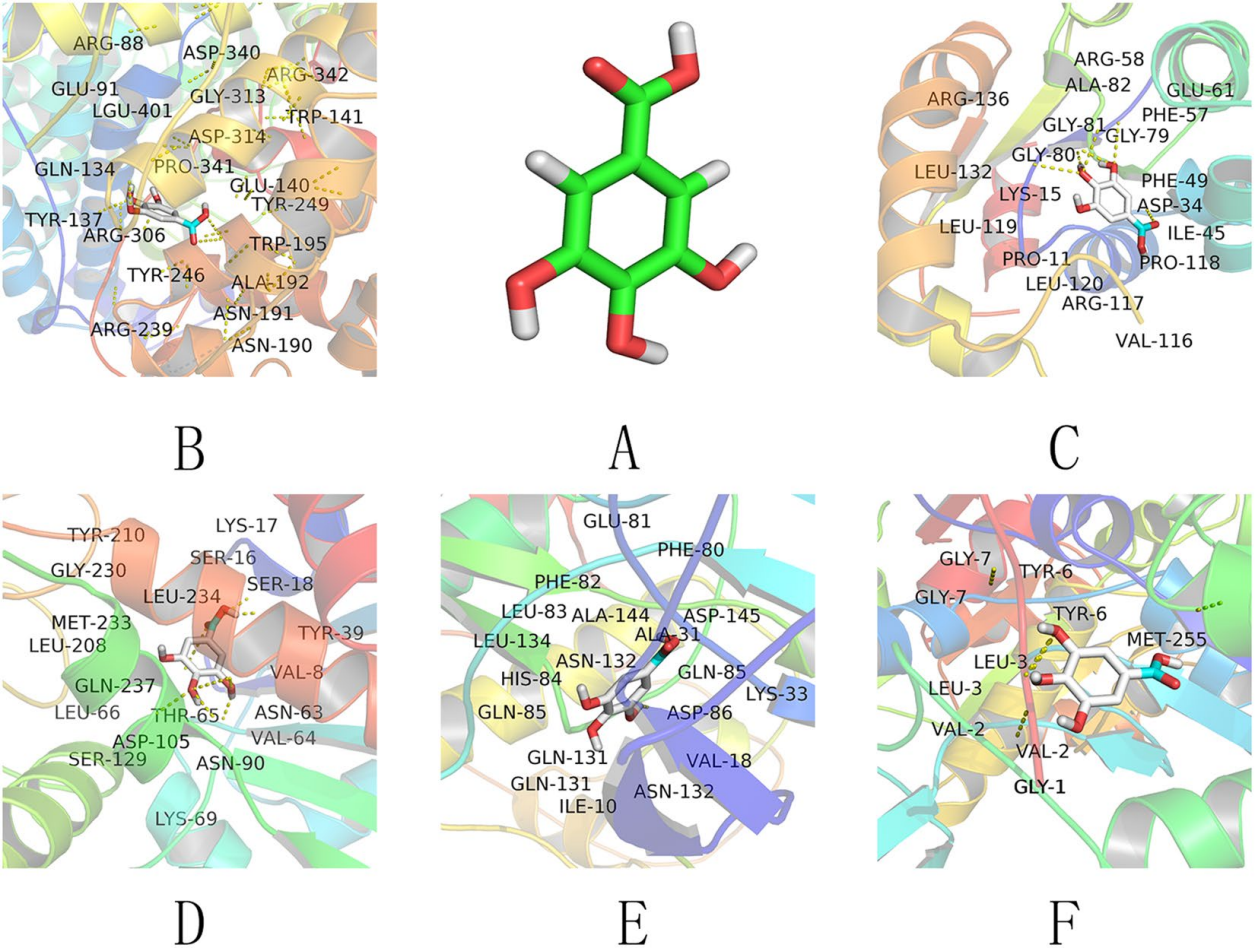
Molecular docking results. (**A**) Tannin 3D mode. (**B**) Docking results for tannin and ALG3. (**C**) Docking results for tannin and ATR. (**D**) Docking results for tannin and ASPM. (**E**) Docking results for tannin and CDKN3. (**F**) Docking results for tannin and BLM. Hydrogen bonds are represented as yellow dashes.

The results of the molecular docking analysis of tannin and the related proteins were as follows [Fig. 9]. The mechanism of ALG3 is to inhibit lymphocyte activation after antigen recognition and exert cytolytic effects on lymphocytes via the complement system. ASPM likely plays a role in mitotic spindle regulation and the coordination of mitotic processes and may play a preferential role in regulating neurogenesis. ATR is also known as high-energy phosphatase and releases energy via hydrolysis to drive cellular chemical reactions. BLM is a solution of the spiral enzyme, which transforms the chemical energy in the cell into mechanical energy to unlock double-stranded DNA. CDKN3 may play a role in regulating. Tannins affect the action of these related proteins and thus play a role in anticancer activity. Docking scores are listed in the Table 2.

**Table 2:** Molecular docking results. Docking results for tannin and ALG3, ASPM, ATR, BLM, CDKN3.

Mode	1	2	3	4	5	6	7	8	9
ALG3 affinity (kcal/mol)	−4.9	−4.9	−4.4	−4.4	−4.4	−4.3	−4.2	−4.1	−3.8
ASPM affinity (kcal/mol)	−6.2	−6.2	−5.9	−5.9	−5.7	−5.6	−5.6	−5.5	−5.5
ATR affinity (kcal/mol)	−5.6	−5.6	−5.5	−5.5	−5.5	−5.5	−5.4	−5.4	−5.2
BLM affinity (kcal/mol)	−6.1	−6.1	−5.4	−5.3	−5.3	−5.3	−5.0	−4.8	−4.5
CDKN3 affinity (kcal/mol)	−5.8	−5.8	−5.7	−5.7	−5.7	−5.7	−5.7	−5.7	−5.7

## Discussion

Cancer is a generalized disease that occurs in a 3D microenvironment and affects the proliferation, cycle, and interaction of cells, as well as the expression of related factors. Among other types of cancer, cervical cancer is can significantly affect the health of women^20,21^. In this study, we used LC-MS, 3D culture, microfluidic chip, flow cytometry, bioinformatics integration and molecular docking technology. We applied tannin extracted from Spatholobi Caulis to cells, and the experimental results show that TTS has a good therapeutic effect against cervical cancer. Microfluidic chip technology has great advantages and potential for the study of TCM. 3D culture systems more closely represent the physiological state *in vivo* than 2D cultures. Compared to our previous 2D system, in 3D culture, drugs are transported by a peristaltic pump into the matrix surrounding the tumor cell aggregates, demonstrating the potential of this 3D system for drug screening in a more physiologically relevant environment. This study lays the foundation for the clinical treatment of cervical cancer with TTS by using a strategy that realized a cell mass fully simulating the growth of cancer cells in the human body. This is the first study to use this 3D microfluidic chip for Spatholobi Caulis-related research. On the one hand, the chip used in this study is designed in four layers, which is good for the formation and growth of cell clusters. Through relevant staining techniques, tumor cell mass growth can be observed and used to determine the dosing time. Each channel has 5 chambers to avoid repeated testing, which is conducive to completing the work and reducing operational errors. Sensitive valve devices can accurately control the opening and closing of the relevant channels. In the meantime, using a syringe pump can reduce reagent consumption via delivery at a rate of 0.2 µL · min^−1^. After 36 hours of treatment, the total amount of reagent use did not reach 0.5 mL. Thus, this approach is more economical than the traditional technique of growing cells in 96-well plates. On the other hand, 3D microfluidic systems can provide a bionic experimental environment representing the human microenvironment^22–24^. By the fluorescent staining of cell smears, the rapid dissociation of dense cancer cell aggregates by the action of the drugs could be observed. Therefore, TTS showed efficacy against cervical cancer similar to that of paclitaxel, the positive control drug.

Using UPLC-Q-TOF/MS, we detected 14 tannin-like components and proved that they are the main active ingredients, laying a material foundation for their use. Cell apoptosis and necrosis can be used to fully determine the efficacy of TTS. FlowJo software was used to analyze the experimental results, and the percentage of apoptosis increased with increasing dose. TTS showed an effect similar to that of the positive control drug, as well as obvious efficacy in promoting apoptosis and necrosis. The cell cycle can reflect the specific period during which the drug inhibits cell division. By ModFit analysis of the cell cycle, TTS was found to arrest cells in the G0/G1 phase, effectively reducing protein and DNA synthesis and inhibiting cell division. Using the GEO and TCGA databases in the gene chip analysis, we found the same differentially expressed genes. The identification of carcinogenesis-related RNAs in this study may have important clinical significance. First, some carcinogenesis-related RNAs were identified in silico, expanding the scope of studied RNAs that are associated with cervical cancer patient outcome. Second, these RNAs can be applied in the evaluation of cervical cancer characteristics. The clinical examination of these RNAs may be beneficial for comprehensive disease management. Third, upregulated and downregulated RNAs in cervical cancer were obtained via high-throughput sequencing. We identified the gene interactions by GO function and KEGG pathway analyses and found clear pathways related to cancer genes, while protein-protein network analysis identified the relationships among proteins. Hence, the disadvantages of analyzing a single protein were avoided. Molecular docking experiments with tannin were performed using the related protein binding sites to validate the five states evaluated for the proteins. The docking simulation successfully revealed the experimental binding mode of tannin with the proteins.

This study shows that the designed chip is conducive to the formation of cell clusters, which can be applied to the detection of drug efficacy. The components contained in the tested drug were determined by UPLC-Q-TOF/MS, which laid the material foundation for the therapeutic use of TTS. In summary, TTS successfully inhibited the proliferation HeLa cervical cancer cells, promoted cell mass shrinkage and reduced the intercellular force, thus reducing cell viability. In addition, TTS not only promoted the apoptosis and necrosis of HeLa cells but also delayed the proliferation of HeLa cells by inhibiting protein and DNA synthesis during the cell division, according to the clinical data derived from the gene chip and molecular docking analyses. We successfully identified the effect of tannin on the relevant genes and proteins. This experiment provides new avenues for the use of TTS in treating cervical cancer, a theoretical basis for the research and development of new drugs, and a basis for the development and utilization of Spatholobi Caulis.

TTS, as a group of compounds extracted from Spatholobi Caulis, showed greater antitumor activity than that found in previous studies, and the composition of TTS has been demonstrated to involve a number of compounds, including methyl gallate, gallic acid, β-glucogallin, procyanidin B1, and catechin. Using this 3D microfluidic chip device and the resulting data, we can conclude that TTS inhibits the proliferation and promotes the apoptosis of HeLa cervical cancer cells. Furthermore, advanced gene chip and molecular docking technology should be used to further develop the efficacy of TTS by providing additional evidence of the anticancer action of TTS, as well as a clear chemical composition; TTS has the potential to be developed into a new anticancer drug from the molecular level.

## Methods

### Extract preparation

Samples were prepared as follows: The herbs were ground and passed through a 40 mm mesh sieve. Samples accurately weighed to 50 g were placed into a round-bottom flask and subjected to extraction twice with 10 times the amount of 60% ethanol solution (1 hour each). After the filtrate was combined, the final drug concentration was adjusted to 0.20 g · mL^−1^ (m/v). This solution was subjected to extraction twice with an equal volume of ethyl acetate; then, the ethyl acetate layer was evaporated.

### Component analysis

An Agilent 6550 iFunnel Q-TOF system was used for chromatography in the experiment. The sample concentration was 8 µg/mL, and the injection volume was 0.2 µL. The separation was performed on a 2.1 · 100 mm Poroshell 120 SB-C18 column (Agilent, USA). The mobile phases were composed of 0.1% formic acid in water (solvent B) and acetonitrile (solvent A). The flow rate was set at 0.6 mL · min^−1^. This method was used for gradient elution^25–27^.

### Cell culture

HeLa cervical cancer cells were obtained from the Cell Bank of Type Culture Collection of Chinese Academy of Sciences. Cells were cultured in basic Dulbecco’s modified Eagle’s medium (DMEM, Gibco, Thermo Fisher Scientific) containing 10% fetal bovine serum in an incubator containing 5% carbon dioxide (Nuaire, USA) at 37 °C. Upon reaching approximately 80% confluence, the cells were digested with 0.25% pancreatin (Trypsin-EDTA, Gibco, Grand Island, USA) and placed in a single-cell suspension for the subsequent processes^28^.

### Microfluidic chip fabrication

**Figure 10:**
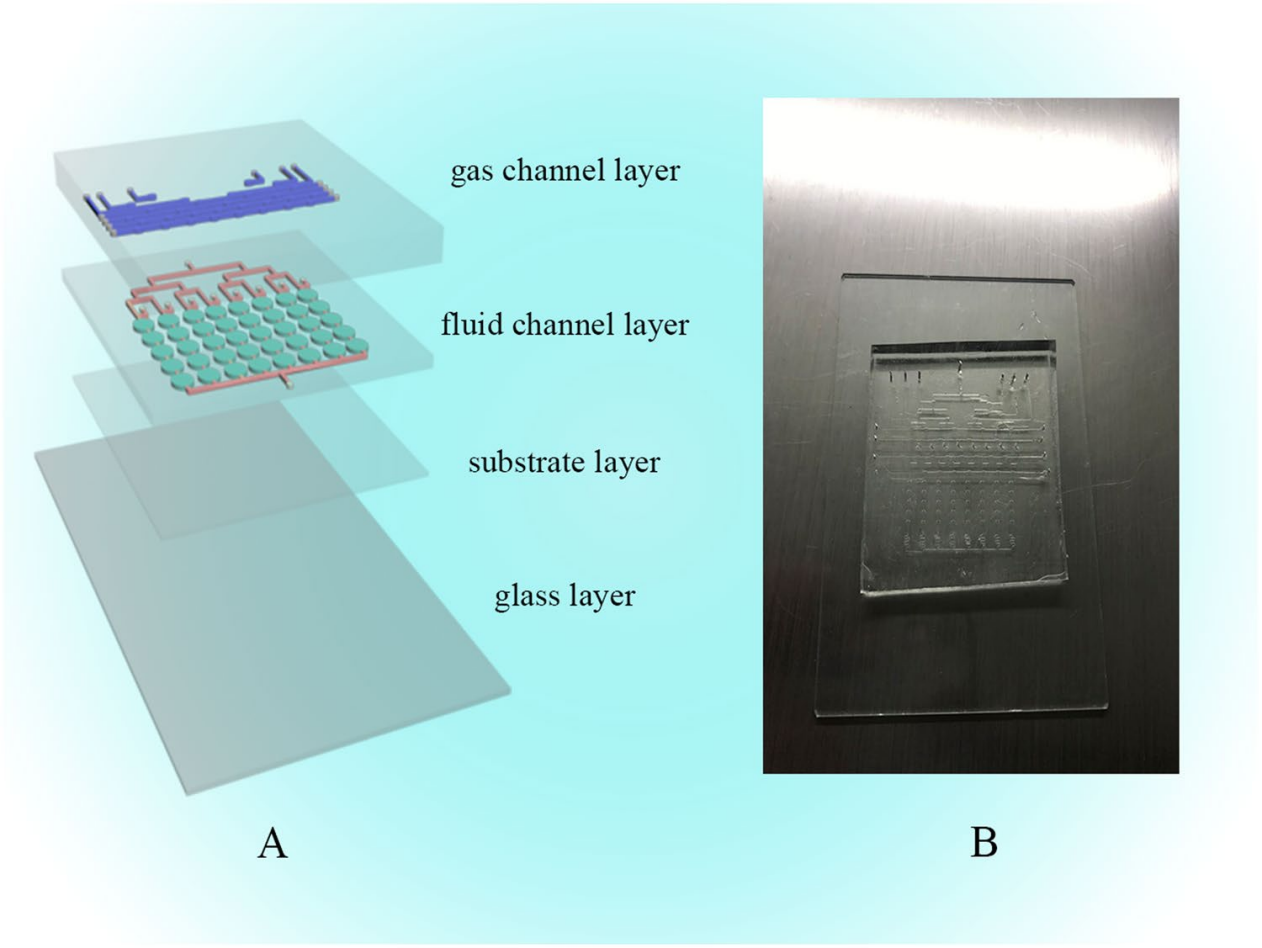
Design of the microfluidic cell culture chip. (**A**) Schematic of the microfluidic chip with the GCL and FCL. The blue channels are in the GCL, and the red channels are in the FCL. The green ellipses are cell growth chambers. (**B**) Image of the chip.

The design of the 3D microfluidic device consists of three layers of polydimethylsiloxane (PDMS, Dow Corning, Midland, MI, USA) and one layer of glass. We designed a system consisting of a gas valve (GV) and a liquid valve (LV). The first layer was designed as a gas channel layer (GCL), the second layer was designed as a fluid channel layer (FCL), the third layer was designed as a substrate, and the last layer was glass. All four layers were combined together by oxygen plasma exposure for irreversible chemical bonding. A schematic diagram of this chip is shown in Fig. 10A. The chip was manufactured by the classic soft lithography method. First, the glass and the silicon template were ultrasonically cleaned with ethanol and acetone, and then the water on the surface was dried initially with nitrogen and subsequently with a heating platform at 105 °C. SU8-2075 negative photoresist (MicroChem, Newton, MA, USA) was spin-coated onto the silicon and patterned by photolithography. After the baking process, the chip model was fabricated. Second, the FCL and basal layer were made by mixing PDMS with hardener (Sylgard 184, Dow Corning, Midland, MI, USA) at a ratio of 15:1, yielding the FCL with 8 liquid channels. The GCL was made by a similar process with mixing at a ratio of 8:1 (PDMS:hardener). The FCL had 10 gas channels. All the mixtures were degassed in a vacuum oven to prevent air bubbles from interfering. Each layer of the chip was formed by pouring or spin coating these colloids and baking at the programmed temperature for curing. After peeling the resist off the master, the PDMS layer was trimmed and punched. The resulting PDMS structure and glass were oxidized in oxygen plasma for 3 minutes for chemical bonding [Fig. 10B]^29–31^. In the GCL, an elliptical gas configuration of 1.00 mm (length) × 0.50 mm (width) × 0.05 mm (height) was fabricated. Each cell culture module in the FCL was approximately 1.50 mm (length) × 1.00 mm (width) × 0.10 mm (height) in size.

### Cell growth activity in the chip

A cell suspension containing 1% sodium alginate (Sigma-Aldrich, USA) and a culture solution containing 1% calcium chloride (Sigma-Aldrich, USA) were prepared. Then, they were injected into the chip for 3D gel culture using a peristaltic syringe pump (Longer Pump, LSP04-1A, China). Cell morphology was observed after 3, 6, 9 and 12 days. Medium containing 1% sodium citrate was injected into the chip using a peristaltic syringe pump to dissolve the colloid, and the cells were collected to prepare a cell smear. Wright-Giemsa (Solarbio, USA) reagent staining was used to observe the state of the cell mass^32^.

### TTS efficacy studies in chip

After 12 days of culture, the cells were divided into low-dose, medium-dose, high-dose, and control groups. After 36 hours, the cells were dissolved in 1% sodium citrate solution, collected and stained with fluorescent dye. Calcein AM and propidium iodide (PI) are two fluorescent dyes that are used to detect live and dead cells, respectively. A volume of 5 μL of an 8 mM PI stock solution and 5 μL of a 4 mM calcein AM stock solution were added to 10 mL of sterile, tissue culture-grade D-PBS, and the solution was vortexed to ensure thorough mixing. The resulting working solution was used to stain the cells by incubation for 40 minutes at room temperature. Then, the samples were washed three times in phosphate-buffered saline (1× PBS). The prepared cell smears^33^ were observed and imaged using an inverted fluorescence microscope (Nikon ECLIPSE TI, Nikon, Japan), and the cell viability (%) was calculated using Image-Pro Plus (IPP) software.

### Flow cytometry analysis of apoptosis and the cell cycle

The cell suspension was adjusted to a density of 5 × 10^5^ mL^−1^, and the cells were seeded in 6-well plates. When the tumor cell density approached 80%, TTS at 0.00, 0.50, 1.00, 2.00 mg · mL^−1^ and a positive control drug (paclitaxel, 6 µg · mL^−1^, Sichuan, China) were added to the wells for 36 hours. After 36 hours, the percentage of apoptotic cells was determined using an Annexin V-FITC Apoptosis Detection Kit (KeyGEN BioTECH, Nanjing, China). The cells were collected, and the samples in each group were added to 500 µL of 1x Binding Buffer, 5 μL of Annexin V-FITC and 5 μL of PI. Flow cytometry (BD Accuri C6, USA) and FlowJo software were used to analyze apoptosis^34^. A Cell Cycle Detection Kit (KeyGEN BioTECH, Nanjing, China) was used to assess cell cycle status. After the cells were collected, they were incubated with 500 µL of 70% cold ethanol for 12 hours at 4 °C. Then, 500 µL of 1x Binding Buffer, 5 µL of RNase A, 5 µL of PI and 1 µL of Triton X-100 were added, and the samples were incubated for 30 minutes in the dark at room temperature for subsequent analysis by flow cytometry (BD Accuri C6, USA) using ModFit software^35^.

### Bioinformatics analysis

Gene expression levels reported for 309 individuals (306 cervical cancer patients and 3 healthy subjects) included in the TCGA database (https://cancergenome.nih.gov/) were integrated using Perl. A matrix describing differentially expressed RNAs was generated using the edgeR package (Bioconductor software). To select for differentially expressed RNAs in the GEO database (http://www.ncbi.nlm.nih.gov/geo), Series GSE63514, which contains 28 cervical cancer tissues and 24 normal tissues, was analyzed and selected using R with the limma package (Bioconductor software). The RNAs were functionally analyzed by KEGG, GO, and protein-protein interaction network analyses^36^.

### Molecular modeling

AutoDock tools and other software were used for molecular modeling. We carefully designed and investigated the binding modes of our compounds against related protein targets through molecular docking studies. A widely adopted structure-based drug design strategy was used to predict the binding modes and affinity of the protein-ligand complexes.

### Statistical analysis

One-way ANOVA was used to test for statistical significance for all *in vitro* experiments using SPSS sofware (SPSS Inc., Chicago). Values with **p < 0.01 were considered statistically significant. Data are presented as the mean (standard deviation).
